# Discovery of *CCDC188* gene as a novel genetic target for human acephalic spermatozoa syndrome

**DOI:** 10.1093/procel/pwae018

**Published:** 2024-04-15

**Authors:** Jing Wang, Hui-Juan Jin, Yi Lu, Zi-Han Wang, Teng-Yan Li, Lan Xia, Hong-Jun Li, Bin-Bin Wang, Su-Ren Chen

**Affiliations:** Department of Medical Genetics and Developmental Biology, School of Basic Medical Sciences, Capital Medical University, Beijing 100069, China; Key Laboratory of Cell Proliferation and Regulation Biology, Ministry of Education, Department of Biology, College of Life Sciences, Beijing Normal University, Beijing 100875, China; Graduate School, Chinese Academy of Medical Sciences & Peking Union Medical College, Beijing 100005, China; Department of Urology, Peking Union Medical College Hospital, Beijing 100005, China; Department of Medical Genetics and Developmental Biology, School of Basic Medical Sciences, Capital Medical University, Beijing 100069, China; Center for Genetics, National Research Institute of Family Planning, Beijing 100081, China; NHC Key Laboratory of Reproductive Health Engineering Technology Research (NRIFP), Beijing 100081, China; Key Laboratory of Cell Proliferation and Regulation Biology, Ministry of Education, Department of Biology, College of Life Sciences, Beijing Normal University, Beijing 100875, China; Graduate School, Chinese Academy of Medical Sciences & Peking Union Medical College, Beijing 100005, China; Department of Urology, Peking Union Medical College Hospital, Beijing 100005, China; Graduate School, Chinese Academy of Medical Sciences & Peking Union Medical College, Beijing 100005, China; Center for Genetics, National Research Institute of Family Planning, Beijing 100081, China; NHC Key Laboratory of Reproductive Health Engineering Technology Research (NRIFP), Beijing 100081, China; Key Laboratory of Cell Proliferation and Regulation Biology, Ministry of Education, Department of Biology, College of Life Sciences, Beijing Normal University, Beijing 100875, China

**Dear Editor,**

Male infertility is a widespread health problem and affects approximately 6%–8% of the male population (Jiao et al., 2021). Acephalic spermatozoa syndrome (ASS), a rare but severe type of teratozoospermia, is characterized by decapitated flagella in the semen and it finally leads to male infertility (Yuan et al., 2015). ASS has been identified to be familial, strongly suggesting that it has a genetic origin. In 2016, the first disease-causing gene of ASS, *SUN5*, is discovered (Zhu et al., 2016). *SUN5* encodes a transmembrane protein specifically expressed in the neck region of sperm, and *Sun5*-KO mice produce headless sperm (Shang et al., 2017). In 2018, the second pathogenic gene of ASS, *PMFBP1*, is identified (Zhu et al., 2018). PMFBP1 is also specifically expressed in the neck region of sperm and *Pmfbp1*-KO male mice are infertility due to the production of acephalic spermatozoa (Zhu et al., 2018). Subsequently, mutations in *SUN5* and *PMFBP1* are reported in more infertile men with ASS by different laboratories, confirming a predominant contribution of these two gene mutations to human ASS (Ekjgatib et al., 2017; Lu et al., 2021; Sha et al., 2018, 2019; Xiong et al., 2022). However, approximately 30% of infertile men with ASS could not be explained by mutations in *SUN5* and *PMFBP1*. Accordingly, it is important to explore more reliable inherited factors for ASS, which will expand the causative gene spectrum of ASS and provide fertility guidance for ASS patients.

It is widely accepted that ASS results from defects in the formation and stability of the head–tail coupling apparatus (HTCA) in the sperm neck region. Ultrastructural studies reveal that HTCA has a structure lining the implantation fossa of the nucleus called the basal plate (Bp) and a region conforming to the concavity of the Bp called the capitulum (Cp). Extending backward from the Cp are nine cylindrically segmented columns (Sc). Proximal centriole (Pc) is also a structural element of HTCA and it is enclosed in a cylindrical niche beneath the Cp (Wu et al., 2020). By ultrastructural observations of HTCA, ASS is classified into three subtypes accordingly to the broken points. The breakage between two centrioles is defined as subtype I of ASS; the broken site of subtype II is between the nucleus and the proximal centriole; the broken point of subtype III is between the distal centriole and midpiece (Wang et al., 2022). Although the structure of HTCA has been described, its molecular compositions, assembly properties, and developmental mechanisms are largely unknown.

In this study, a homozygous nonsense variant in *CCDC188* is identified in an ASS patient (Fig. 1A and Table S1). The ASS phenotype of the proband was illustrated by Papanicolaou staining, scanning electron microscope (SEM), and transmission electron microscope (TEM) of spermatozoa (Figs. 1B–D and S1A). ASS in the proband matched the phenotypes of subtype II of ASS, of which the broken point was between the nucleus and the proximal centriole. No variations in *SUN5* and *PMFBP1*, two recognized ASS-causing genes, were identified. Accordingly, WES was performed to explore the genetic cause of ASS using the DNA samples from the proband (IV-1) and his parents (III-3 and III-4). Homozygous variants in 14 genes, compound heterozygous variants in 2 genes, and hemizygous in 1 gene were screened out following the criteria (Fig. S1B). A homozygous nonsense variant c.937C>T/p.R313* in the *CCDC188* gene caught our attention because this functional unknown protein is specifically expressed in testes. Other 16 candidate genes (Table S2) do not show a clear relationship with male fertility. Sanger sequencing of *CCDC188* revealed that the proband carried a homozygous nonsense variant, and his consanguineous parents harboured a heterozygous allele (Fig. 1E). This variant was not found in the public databases and was predicted to be harmful to protein’s function (Table S2).

**Figure 1.**
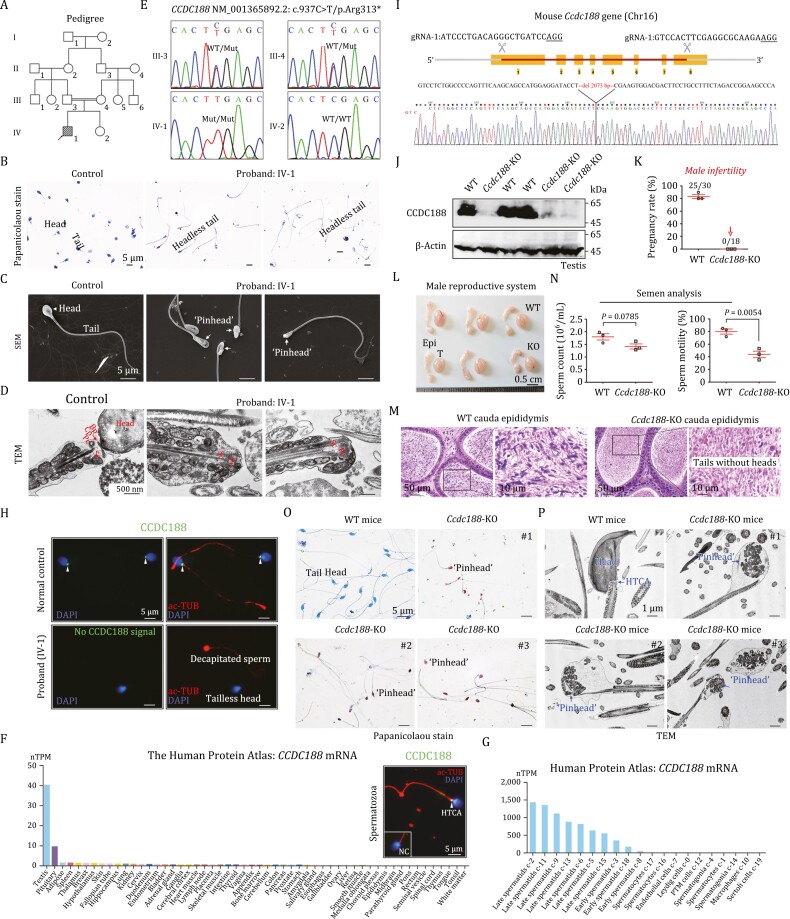
**Identification of a homozygous nonsense variant in the *CCDC188* gene in an infertile patient with ASS and generation of *Ccdc188*-KO mice.** (A) Pedigree of the recruited patient’s family. Black-filled square indicates the proband (IV-1). (B) Sperm morphology of the proband and a normal control was examined by Papanicolaou staining. Scale bars, 5 μm. (C) SEM was performed to reveal the ‘pinhead’ structure (arrows) in the front of the headless flagella in the proband. Scale bars, 5 μm. (D) TEM analysis indicated the ultrastructure of HTCA of sperm from the proband and a normal control. Bp, basal plate; Cp, capitulum; Pc, proximal centriole; Sc, segmented column. Scale bars, 500 nm. (E) Sanger sequencing validation of the variant of *CCDC188*, c.937C > T/p.Arg313*, in the proband and all available family members. (F) Among different human tissues, *CCDC188* mRNA was predominantly expressed in the testes, according to the Human Protein Atlas. Immunofluorescence staining of CCDC188 and acetylated Tubulin (ac-TUB) in a human spermatozoon. Scale bar, 5 μm. NC, negative control, IgG was used. (G) In human testes, *CCDC188* mRNA was restricted to early/late spermatids, according to the Human Protein Atlas. (H) Immunofluorescence staining of CCDC188 on spermatozoa from the proband and a normal control. Flagella and nucleus were stained with ac-TUB and DAPI, respectively. Arrowheads indicated CCDC188 signals. Scale bar, 5 μm. (I) Schematic illustration of the targeting strategy for generating *Ccdc188*-KO mice by using CRISPR/Cas9 technology. gRNA sequences and Sanger sequencing were provided. (J) Immunoblotting of CCDC188 was performed in the testis protein lysates of WT mice and *Ccdc188*-KO mice. β-Actin was used as an internal control. (K) Fertility assessment experiments were performed on three adult *Ccdc188*-KO male mice and three WT male littermates for 2 months. (L) Reproductive system of adult WT mice and *Ccdc188*-KO mice. T, testis; Epi, epididymis. Scale bars, 0.5 cm. (M) Histological morphology of cauda epididymis from WT mice and *Ccdc188*-KO mice by haematoxylin-eosin (H&E) staining. Scale bars, 50 or 10 µm. (N) Sperm counts were counted with a fertility counting chamber under a light microscope and total motility was assessed by a CASA system. Data are presented as the mean ± SD (*n *= 3 each group), Student’s *t* test. (O) Morphological analyses of sperm in WT mice and *Ccdc188*-KO mice by Papanicolaou staining. Arrows indicated the pinheads. Scale bars, 5 μm. (P) TEM images of sperm from WT mice and *Ccdc188*-KO mice. Scale bars, 1 μm.

CCDC188 is a functionally unknown protein. The mouse *Ccdc188* is orthologous to human *CCDC188* (protein orthologs 89.66%) (Fig. S2A), and they show restricted expression towards testis (spermatids/sperm subpopulation) according to the database (Figs. 1F, 1G and S2B). Using CCDC188 antibody generated by our laboratory, we showed that CCDC188 was specifically localized at the HTCA of human and mouse sperm (Figs. 1F and S2B). The HTCA-specific expression of CCDC188 in human and mouse sperm indicates that CCDC188 may regulate the sperm head–tail linkage. Expressions of SUN5 and PMFBP1 could be detected in sperm samples from either the proband or a fertile normal control, whereas CCDC188 was absent in sperm protein lysates from the proband (Fig. S2C). Immunofluorescence staining of the proband’s sperm also indicated that CCDC188 signals could not be detected in both the tailless heads and the acephalic tails (Fig. 1H). Thus, ASS phenotype of the proband is most likely caused by the homozygous nonsense variant of *CCDC188*.

To explore the physiological role of CCDC188, we produced *Ccdc188*-KO mice by applying CRISPR/Cas9 technology (Fig. 1I). By Western blot analysis, CCDC188 expression was confirmed to be absent in the testis of *Ccdc188*-KO mice (Fig. 1J). *Ccdc188*-KO mice were healthy and developed normally; however, the fertility test showed that *Ccdc188*-KO male mice were completely infertile (Fig. 1K). The testis size was similar between *Ccdc188*-KO mice and wild-type (WT) mice (Fig. 1L). Histological examination of testis sections revealed generally normal spermatogenesis in *Ccdc188*-KO mice; however, abnormalities could be identified at stages V–VIII of seminiferous epithelium, in which sperm inside the lumen were tailless (Fig. S3A). Histological examination of caudal epididymis showed that sperm from *Ccdc188*-KO mice were not stained by haematoxylin, indicating the absence of sperm heads (Fig. 1M). Semen characteristics were further analysed between *Ccdc188*-KO mice and WT mice. Sperm count showed a slight but not significant reduction whereas sperm motility was significantly attenuated in *Ccdc188*-KO mice (Fig. 1N and Movies 1, 2). As revealed by Papanicolaou staining, sperm extracted from caudal epididymis all lacked of heads in *Ccdc188*-KO mice, and the ‘pinhead’ was present at the front of headless flagella (Figs. 1O and S3B). Ultrastructure examination revealed that headless sperm tails of *Ccdc188*-KO mice contained a residual droplet of cytoplasm at the top of the flagella with misarranged mitochondria inside (“pinhead”) (Figs. 1P and S3C). The ultrastructure of acrosome (in the separated heads) and axoneme (in headless flagella) from *Ccdc188*-KO mice was generally normal (Fig. S3C).

Given that SUN5 and PMFBP1 are two recognized ASS-causing proteins, we examined their expression in the testis protein lysates and found that their protein levels were not altered after the loss of CCDC188 (Fig. 2A and 2B). Immunofluorescence staining was subsequently performed using sperm from mice and humans. In WT mice and a normal male individual, both SUN5 and PMFBP1 were localized at the HTCA of the intact sperm (Fig. 2C–F). In *Ccdc188*-KO mice and the proband (IV-1), heads and tails of sperm were separated from each other. SUN5 signals were visible at the end of sperm heads (implantation fossa) and PMFBP1 was distributed at the front of sperm tails ("pinhead") (Fig. 2C–F). The fracture occurs between SUN5 and PMFBP1, indicating that CCDC188 may mediate their interaction.

**Figure 2.**
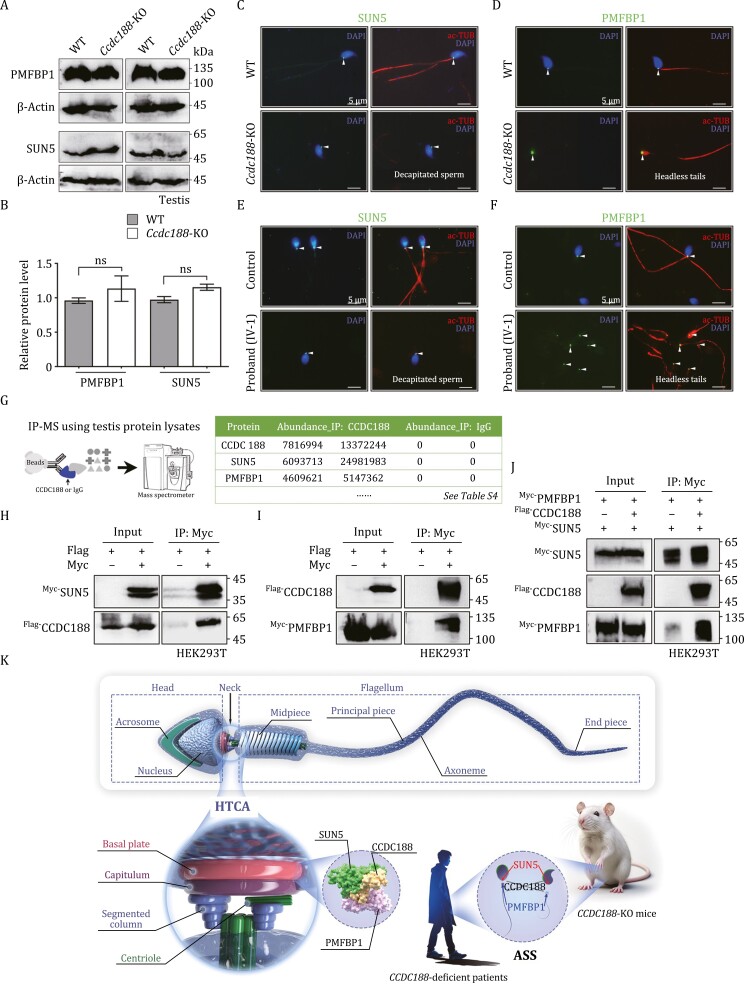
**CCDC188 cooperates with SUN5 and PMFBP1 and mediates their interaction.** (A) Immunoblotting of PMFBP1 and SUN5 was performed in the testis protein lysates of WT mice and *Ccdc188*-KO mice. (B) Relative protein levels of PMFBP1 and SUN5 as analysed of blots in A. Data are presented as the mean ± SD; Student’s *t* test; ns, not significant. (C and D) Immunofluorescence staining of SUN5 (C) and PMFBP1 (D) on sperm from WT mice and *Ccdc188*-KO mice. Flagella and nucleus were stained with ac-TUB and DAPI, respectively. Scale bar, 5 μm. (E and F) Immunofluorescence staining of SUN5 (E) and PMFBP1 (F) on sperm from the proband and a normal control. Scale bar, 5 μm. Arrowheads in (C–F) indicated the signals of SUN5 or PMFBP1. (G) Testicular interaction components of CCDC188 were identified by IP-MS (using anti-CCDC188 antibody or anti-IgG antibody). SUN5 and PMFBP1 were identified as potential CCDC188-interacting proteins and all candidates were listed in Table S3. (H) Flag-tagged CCDC188 could be immunoprecipitated by Myc-tagged SUN5 in HEK293T cell extracts. (I) Myc-tagged PMFBP1 was immunoprecipitated by Flag-tagged CCDC188 in HEK293T cells. (J) Flag-tagged CCDC188 was transfected into HEK293T cells with Flag-tagged PMFBP1 and Myc-tagged (or Flag-tagged CCDC188 alone). Anti-Myc antibody was utilized to immunoprecipitate Myc-tagged SUN5. Flag-tagged PMFBP1 was only immunoprecipitated by Myc-tagged SUN5 in HEK293T cells when Flag-tagged CCDC188 was co-transfected. (K) Schematic diagram illustrating the role of CCDC188 in sperm head and tail connection. CCDC188 connects SUN5 and PMFBP1. Knockout of *Ccdc188* in mice or loss-of-function mutation of *CCDC188* in humans leads to the HTCA broken and separation of SUN5 and PMFBP1 into corresponding two parts.

To explore the interactome of CCDC188 and shed light on the regulatory role of CCDC188 in head–tail connection, we performed immunoprecipitation-mass spectrometry (IP-MS) in mouse testis protein lysates by using CCDC188 antibody or IgG antibody (two independent replicates for each group) (Fig. 2G). Intriguingly, SUN5 and PMFBP1, two well-known ASS-associated proteins, were identified as potential CCDC188-binding partners (Fig. 2G). The full list of proteins enriched (fold change > 10) in the CCDC188-IP group (as compared with the IgG-IP group) was provided in Table S3. The interaction between Flag-tagged CCDC188 and Myc-tagged SUN5 (or PMFBP1) was further confirmed by co-immunoprecipitation (co-IP) assays in HEK293T cells (Fig. 2H and 2I). Previous studies find that SUN5 and PMFBP1 does not direct interact with each other (Zhang et al., 2021; Zhu et al., 2018), indicating that some other HTCA components might be present to mediate their connection. We further showed that Flag-tagged PMFBP1 could be immunoprecipitated by Myc-tagged SUN5 in the case of co-expression of Flag-tagged CCDC188 in HEK293T cells (Fig. 2J), indicating that CCDC188 may be one of the missing linkers between SUN5 and PMFBP1 in sperm HTCA.

In summary, we identified a homozygous nonsense variant, c.937C > T/p.R313*, in *CCDC188* gene using WES in a consanguineous family with an infertile proband suffering from ASS. CCDC188 was specifically expressed at the HTCA of human and mouse sperm and *Ccdc188*-KO mice exhibited male infertility with a typical ASS phenotype. At molecular levels, CCDC188 interacted with SUN5 and PMFBP1 and mediated their interaction. Absence of CCDC188 set SUN5 and PMFBP1 apart, the former in the sperm head and the latter in the decapitated tail. These results suggest that CCDC188 is critical for mammalian head–tail connection and *CCDC188* gene could be further developed as genetic markers for diagnosing ASS in humans (Fig. 2K).

Our current study discovered *CCDC188* as a novel ASS-causing gene in humans and mice; however, evidence is still needed to test and verify the genetic contribution of *CCDC188* variants to ASS in humans. We are recruiting larger cohorts of ASS patients without mutations in *SUN5* and *PMFBP1*. Furthermore, examination of *CCDC188* mutations in infertile men with ASS by different clinical centres is also very helpful to promote the clinical application of *CCDC188* variant detection to the diagnosis of ASS. A previous study suggests that Centlein also mediates the connection between SUN5 and PMFBP1 (Zhang et al., 2021). Thus, it will be interesting to study the relationship between Centlein and CCDC188 in the HTCA assembly. In future, structural biology will also provide direct evidence to explore the binding information of ‘SUN5-CCDC188-PMFBP1’ complex in sperm HTCA assembly.

Intracytoplasmic sperm injection (ICSI) has been suggested to be useful for infertile men with ASS if acephalic sperm heads are carefully selected to perform ICSI (Shang et al., 2017). Unfortunately, the proband in our study is unmarried; accordingly, the outcomes of ICSI treatment could not be reported in this study. Two-cell embryos and blastocysts were successfully obtained after ICSI using the acephalic sperm heads in the semen of *Ccdc188*-KO mice (Fig. S3D). Based on our attempts in mice, we suggest that *CCDC188* mutation-associated infertility in humans is likely overcome by ICSI treatment.

Materials and methods, primers for Sanger sequencing (Table S4), sgRNA sequences to generate knockout mice (Table S5), primers for plasmid construction (Table S6), and antibodies used in this study (Table S7) were provided in the supplementary information.

## Supplementary information

The online version contains supplementary material available at https://doi.org/10.1093/procel/pwae018.

pwae018_suppl_Supplementary_Material

pwae018_suppl_Supplementary_Video_S1

pwae018_suppl_Supplementary_Video_S2

## Footnotes

We acknowledge Xi-Xia Li and Shuang-Zhong Lv from the Center for Biological Imaging (CBI), Institute of Biophysics, Chinese Academy of Sciences for their help in making TEM samples.

The authors declare their agreement to participate and to publish. They declare no conflict of interests.

This work was supported by the National Natural Science Foundation of China (32370905), the Central Government to Guide Local Scientific and Technological Development (ZY21195023), the National Key Research and Development Project (2019YFA0802101), the Fundamental Research Funds for the Central Institutes (2023GJZD01), the Basic Research Projects of Central Scientific Research Institutes (2023GJZD01), and the Open Fund of Key Laboratory of Cell Proliferation and Regulation Biology, Ministry of Education. The study was conducted in accordance with the principles of the Declaration of Helsinki and approved by the institutional review boards at the Peking Union Medical College Hospital. Signed informed consents were obtained from all subjects participating in the study. Animal experiments were approved by the Animal Care and Use Committee of the College of Life Sciences, Beijing Normal University.

The mass spectrometry data of IP-MS have been deposited to the ProteomeXchange Consortium with the dataset identifier PXD045762. All data generated or analysed during this study are included in this published article (and its supplementary information files), further inquiries can be directed to the corresponding authors.

S.R., B.B., and H.J. conceived and designed the project; J.W. and Y.L. collected samples from humans and performed the W.E.S. and Sanger sequencing; H.J. and L.X. performed the animal experiments and co-IP assays; Z.H. and T.Y. conducted the IP-MS; S.R. wrote the manuscript. All of the authors have read and approved the final manuscript.
