# Supplementary material for: Discovery of CCDC188 gene as a novel genetic target for human acephalic spermatozoa syndrome
Source: Protein Cell. 2024 Apr 15;15(9):704–9. doi: 10.1093/procel/pwae018 (PMC11365554; doi:10.1093/procel/pwae018)
Supplement: pwae018_suppl_Supplementary_Material [file pwae018_suppl_supplementary_material.pdf]

Supplemental Materials

Materials and methods

Study participants

An infertile men, his brother, and his parents were recruited at the Peking Union Medical College Hospital (Beijing, China). The study was conducted in accordance with the principles of the Declaration of Helsinki and approved by the institutional review boards at the Peking Union Medical College Hospital. Signed informed consents were obtained from all subjects participating in the study. The patient exhibited a normal karyotype (46, XY) and normal hormone levels; no microdeletions were found on the Y chromosome. Five milliliters of peripheral blood was collected from the brothers and his parents.

WES and Sanger sequencing

Genomic DNA was isolated from peripheral blood of the subjects via QIAamp DNA Blood Mini Kit (QIAGEN, USA). Agilent SureSelect Human All Exon V6 Kit (Agilent Technologies, USA) was applied to exon capture and Illumina HiSeq X system was utilized to perform sequencing according to the manufacturer's introductions. The average sequencing depth on targets was ~107×, and the ratio of the target fraction covered at minimum 10× was 99.5%. Reads were mapped to the human genome reference (GRCh37/hg19) by the Burrows Wheeler Aligner (BWA) software. ANNOVAR software was utilized for functional annotation via various databases, including dbSNP, 1000 Genomes Project, ExAC, and HGMD. The Genome Analysis Toolkit (GATK 3.7) was employed to identify and quality-filter the variants. Sanger sequencing was applied to verify the mutations detected by WES in the proband, his brother, and his parents. The primers of Sanger sequencing were listed in the Table S4.

Ccdc188-KO mice

Animal experiments were approved by the Animal Care and Use Committee of

the College of Life Sciences, Beijing Normal University. The *Ccdc188* gene (NCBI: XM_011246064.4; Ensembl: ENSMUSG00000090777) has 4 transcripts and 8 exons and is located on mouse chromosome 16. Exons 1~8 of the *Ccdc188-202* (ENSMUST00000231369) transcript were selected as the knockout region. Mouse zygotes were coinjected with an RNA mixture of Cas9 mRNA (TriLink BioTechnologies, CA, USA) and sgRNAs. The sgRNA sequence was provided in Table S5. The injected zygotes were transferred into pseudopregnant recipients to obtain the F0 generation. DNA was extracted from tail tissues from 7-day-old offspring and PCR amplification was carried out with genotyping primers using the Mouse Tissue Direct PCR Kit (Tiangen Biotech, China). The founder mice were mated with wild-type females, giving rise to heterozygous mice harbouring a 2073 bp deletion in the *Ccdc188* gene. A stable F1 generation (heterozygous mice) was obtained by mating positive F0 generation mice with wild-type C57BL/6JG-pt mice.

CCDC188 antibody

Full-length mouse CCDC188 was cloned into the pET-N-His-C-His vector (Beyotime, Shanghai, China) and then transfected into the ER2566 E. coli strain (Weidi Biotechnology, Shanghai, China). Protein expression was induced by 1 mM IPTG (Beyotime) at 30°C overnight. After centrifugation, the bacterial pellet was resuspended in buffer (50 mM Tris-HCl pH 8.0, 200 mM NaCl), and the proteins were released by sonication. After centrifugation, anti-His beads (Beyotime) were added to the supernatant and incubated overnight at 4°C. After washing, recombinant protein was eluted with 250 mM imidazole (Beyotime). Recombinant CCDC188 protein was emulsified at a 1:1 ratio (v/v) with Freund's complete adjuvant (Beyotime) and administered subcutaneously into ICR female mice at multiple points. For the subsequent three immunizations, recombinant CCDC188 protein was emulsified with incomplete Freund's adjuvant (Beyotime) at an interval of 2 weeks. One week after the last immunization, blood was collected, and the serum was separated.

Fertility testing

To confirm the fertility of *Ccdc188*-KO male mice, natural mating tests were conducted. Briefly, adult *Ccdc188*-KO male mice and their littermate wild-type mice (*n*=3 each) were mated with wild-type C57BL/6J females (male: female=1:2) for two months. The vaginal plugs of the mice were examined every morning. Female mice with vaginal plugs were separately fed, and female mice were replenished. The number of pregnant females was recorded.

Semen analysis

Sperm counts were determined using a fertility counting chamber (Makler, Israel) under a light microscope. Sperm mobility was assessed via the application of a computer-assisted sperm analysis (CASA) system (SAS Medical, China). The sperm suspension was mounted on a glass slide, air-dried, and fixed with 4% PFA for 20 min at room temperature. The slides were stained with Papanicolaou solution (Solarbio, Beijing, China) and observed using a DM500 optical microscope (Leica, Germany).

Histological analysis

The testes and caudal epididymis were dissected and fixed in 4% PFA overnight at 4°C. Fixed tissues were embedded in paraffin, sectioned (5 μm thick), dewaxed, and rehydrated. The sections were stained with Periodic Acid Schiff's solution or hematoxylin-eosin staining (H&E) solution before imaging using a Leica DM-500 optical microscope (Leica Microsystems, German).

SEM

Sperm were fixed with 2.5% (vol/vol) glutaraldehyde in 0.1 M phosphate buffer (pH 7.4) at room temperature for 30 min and then deposited on coverslips. The coverslips were dehydrated via an ascending gradient of 50%, 70%, 95%, and 100% ethanol, and air-dried. Specimens were then attached to specimen holders and coated with gold particles using an ion sputter coater before being viewed with a JSM-IT300 scanning electron microscope (JEOL, Tokyo, Japan).

TEM

Sperm were fixed with 2.5% (vol/vol) glutaraldehyde in 0.1 M phosphate buffer (pH 7.4) at 4°C. The samples were washed four times in phosphate buffer and first immersed in 1% (wt/vol) OsO4 and 1.5% (wt/vol) potassium ferricyanide aqueous solution at 4°C for 2 h. After washing, the samples were dehydrated through graded alcohol into pure acetone. Samples were infiltrated in a graded mixture of acetone and SPI-PON812 resin, and then the pure resin was changed. The specimens were embedded in pure resin with 1.5% BDMA, polymerized for 12 h at 45°C and 48 h at 60°C, cut into ultrathin sections (70 nm thick), and then stained with uranyl acetate and lead citrate for subsequent observation and photography with a Tecnai G2 Spirit 120 kV (FEI) electron microscope.

IP-MS

Testes were collected from adult C57BL/6JG-pt mice and lysed with Pierce™ IP Lysis Buffer (Thermo Fisher, CA, USA). Protein lysates were incubated overnight with 2 μg CCDC188 antibody or IgG at 4°C. The lysates were then incubated with 20 μl Pierce™ Protein A/G-conjugated Agarose for 4 h at 4°C. IP products were digested by trypsin digestion for 4 h at 37°C. Separation was performed by a Thermo UltiMate 3000 UHPLC (Thermo Scientific, MA, USA). The peptides separated by liquid phase chromatography were ionized by a nanoESI source and then passed to a tandem mass spectrometer Q-Exactive HF-X (Thermo Fisher Scientific, San Jose, CA) for DDA (Data Dependent Acquisition) mode detection. Protein identification uses experimental MS/MS data and aligns them with theoretical MS/MS data from a database (i.e., UniProt) to obtain results. Mascot 2.3.02 search, quality control, and iBAQ quantification were subsequently performed. The mass spectrometry data have been deposited to the ProteomeXchange Consortium with the dataset identifier PXD045762.

co-IP

HEK293T line was obtained from ATCC (American Type Culture Collection) and authenticated using STR profiling test. HEK293T cells were transfected with Flag and/or Myc-tagged plasmids as indicated in respective figure legends. The primers for plasmid construction were provided in Table S6. Forty-eight hours after transfection, cells were lysed with Pierce™ IP Lysis Buffer (Thermo Fisher, CA, USA) for 30 min at 4°C and then centrifuged at 12,000 × g for 10 min. Protein lysates were incubated overnight with Myc-Tag antibody (2 μg) or Flag-tag antibody (2 μg) at 4°C. The lysates were then incubated with 20 μl Pierce™ Protein A/G-conjugated Agarose for 4 h at 4°C. The agarose beads were washed five times with Pierce™ IP Lysis Buffer and boiled for 5 min in 1×SDS loading buffer. Input and IP samples were analysed by Western blotting using HRP conjugated anti-Flag-Tag or anti-Myc-Tag antibodies. Detailed information of antibodies was provided in Table S7.

Western blotting

Proteins from mouse testes were extracted using RIPA lysis buffer containing 1 mM PMSF and protease inhibitors on ice. Supernatants were collected following centrifugation at 12,000 × g for 10 min. Proteins were electrophoresed in 10% SDS–PAGE gels and transferred to nitrocellulose membranes (GE Healthcare, USA). The blots were blocked in 5% milk and incubated with primary antibodies overnight at 4°C, followed by incubation with anti-rabbit or mouse IgG H&L (HRP) for 1 h. β-Actin served as an internal control. The signals were evaluated using Super ECL Plus Western Blotting Substrate and a Tanon-5200 Multi chemiluminescence imaging system (China). Detailed information of antibodies was provided in Table S7.

Immunofluorescence

After permeabilization with 1% Triton X-100 for 1 h, the slides of sperm were blocked with 5% goat serum for 1 h. Primary antibodies were added and

incubated overnight at 4°C. Detailed information of primary antibodies were provided in Table S7. After washing three times with 1×PBS, slides were incubated with Alexa Fluor 484-labelled or 555-labelled donkey anti-rabbit/mouse IgG (Beyotime, Shanghai, China) for 1 h at room temperature. The nuclei were counterstained with DAPI dye and observed using Leica TCS SP2 microscope.

Statistics

Data are presented as the mean ± standard deviation (SD) and were analysed using GraphPad Prism version 5.01 (GraphPad Software). Student's *t* test (unpaired, two-tailed) was used for the statistical analyses.

Supplemental Figure Legends

Figure S1. Semen analysis of the proband, the flowchart of WES and variant-filtration pipeline. (**A**) Representative images of Papanicolaou staining and scanning electron microscope (SEM) of sperm were shown. Spermatozoa in the semen of the proband showed 100% headless tails. Scale bars, 10 or 5 μm. (**B**) Variants were screened out following the criteria: (1) variants affected coding exons or splice sites (including missense, nonsense, frameshift, and splicing site variants); (2) a minor allele frequency (MAF) was <1% in ExAC for autosomal variants and a MAF was <1‰ in ExAC for variants on the X chromosome; (3) variants were predicted potentially deleterious by at least a pathogenicity prediction software; and (4) variants were consistent with the recessive genetic pattern (homozygous or compound heterozygous) or X-linked inheritance (hemizygous).

Figure S2. Alignment result of human and mouse CCDC188. (**A**) Alignment result of human CCDC188 and mouse CCDC188 was analyzed by M-Coffee website (https://tcoffee.crg.eu/). (**B**) According to the NCBI database, *Ccdc188* mRNA was specifically expressed in the testes among different mouse tissues. According to the Single Cell Expression Atlas, single-cell RNA sequencing of mouse germ cells indicated that *Ccdc188* mRNA was restricted to spermatid subpopulation. Immunofluorescence staining of CCDC188 in a mouse spermatozoon, showing the HTCA-specific localization. For a negative control, IgG was added instead of the primary antibody. Flagella and nucleus was stained with acetylated Tubulin (ac-TUB) and DAPI, respectively. Scale bar, 5 μm. (**C**) The expression of CCDC188, SUN5, and PMFBP1 in sperm samples from the proband and a normal control. β-actin served as a loading control.

Figure S3. More detailed analysis of *Ccdc188*-KO mice. (**A**) Spermatogenesis in *Ccdc188*-KO mice was revealed by Periodic acid-Schiff staining of testis sections. Seminiferous tubules could be divided into I-XII

stages and 16 steps of spermatids could be identified. Scale bars, 50 or 10 μm. (**B**) Papanicolaou stain of sperm smear from WT mice and *Ccdc188*-KO mice. Scale bars, 10 μm. (**C**) Transmission electron microscope (TEM) of sperm from WT mice and *Ccdc188*-KO mice. Scale bars, 500 nm. Nu, nuclear; Acr, acrosome; Ax, axoneme; Mp, midpiece; Pp, principle piece; HTCA, head-tail coupling apparatus; Mt, mitochondrial sheath. (**D**) Intracytoplasmic sperm injection (ICSI) outcomes. *Ccdc188*-KO sperm heads were collected by centrifugation in 70% Percoll (Sigma, P4937) followed by three washes in M2 medium. Single sperm heads were picked up from the sperm suspension and injected into wild-type oocytes using a micromanipulator with a Piezoelectric actuating pipette. 2-cell embryos and blastocysts were shown. Scale bar, 100 μm.

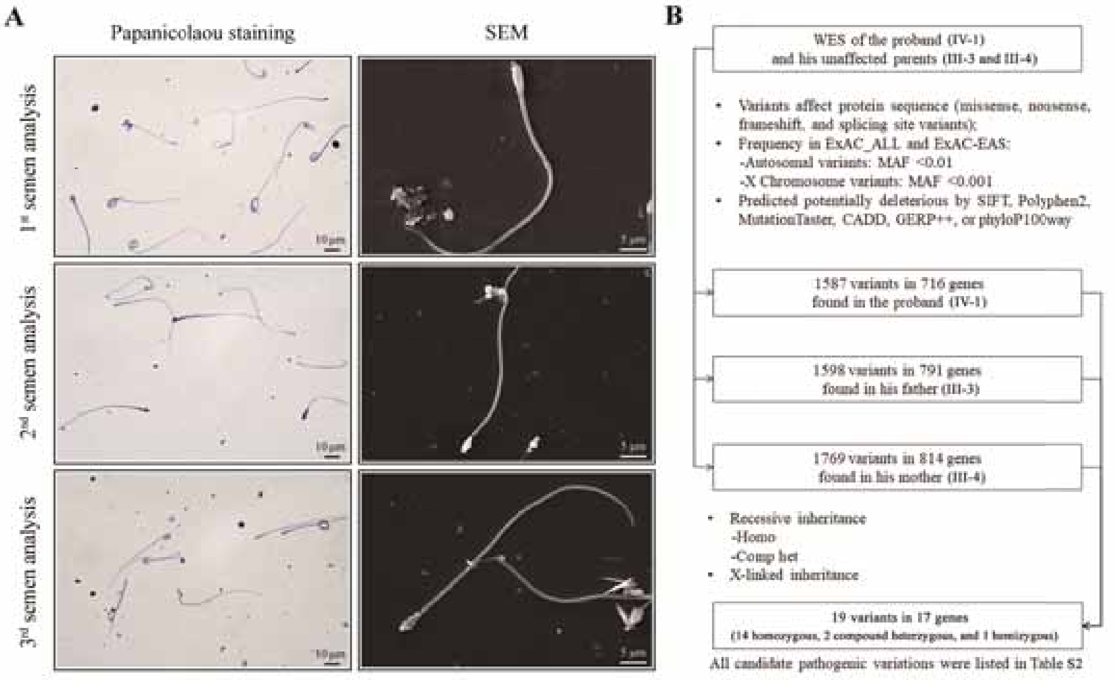
A
Papanicolaou staining
SEM
1st semen analysis
2nd semen analysis
3rd semen analysis
10 μm
5 μm
B
WES of the proband (IV-1)
and his unaffected parents (III-3 and III-4)
• Variants affect protein sequence (missense, nonsense, frameshift, and splicing site variants);
• Frequency in ExAC_ALL and ExAC-EAS:
-Autosomal variants: MAF <0.01
-X Chromosome variants: MAF <0.001
• Predicted potentially deleterious by SIFT, Polyphen2, MutationTaster, CADD, GERP++, or phyloP100way
1587 variants in 716 genes
found in the proband (IV-1)
1598 variants in 791 genes
found in his father (III-3)
1769 variants in 814 genes
found in his mother (III-4)
• Recessive inheritance
-Homo
-Comp het
• X-linked inheritance
19 variants in 17 genes
(14 homozygous, 2 compound heterzygous, and 1 hemizygous)
All candidate pathogenic variations were listed in Table S2

Figure S1.

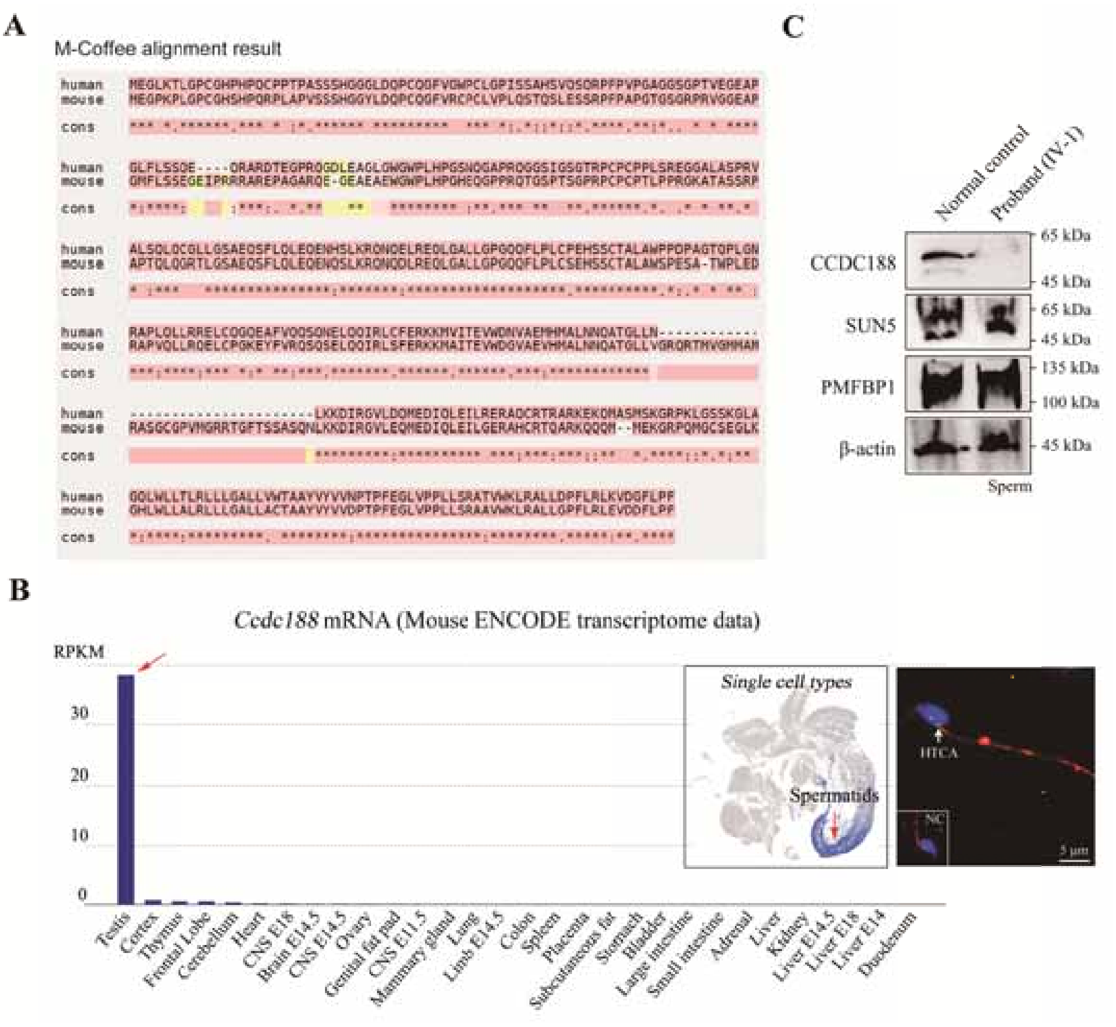
A
M-Coffee alignment result
human
mouse
cons
B
Ccdc188 mRNA (Mouse ENCODE transcriptome data)
RPKM
30
20
10
0
Testis
Cortex
Thymus
Frontal Lobe
Cerebellum
Heart
CNS E18
Brain E14.5
CNS E14.5
Ovary
Genital fat pad
CNS E11.5
Mammary gland
Lung
Limb E14.5
Colon
Spleen
Placenta
Subcutaneous fat
Stomach
Bladder
Large intestine
Small intestine
Adrenal
Liver
Kidney
Liver E14.5
Liver E18
Liver E14
Duodenum
Single cell types
Spermatids
HTCA
NC
5 μm
C
Normal control
Proband (IV-1)
CCDC188
SUN5
PMFBP1
β-actin
65 kDa
45 kDa
65 kDa
45 kDa
135 kDa
100 kDa
45 kDa
Sperm

Figure S2.

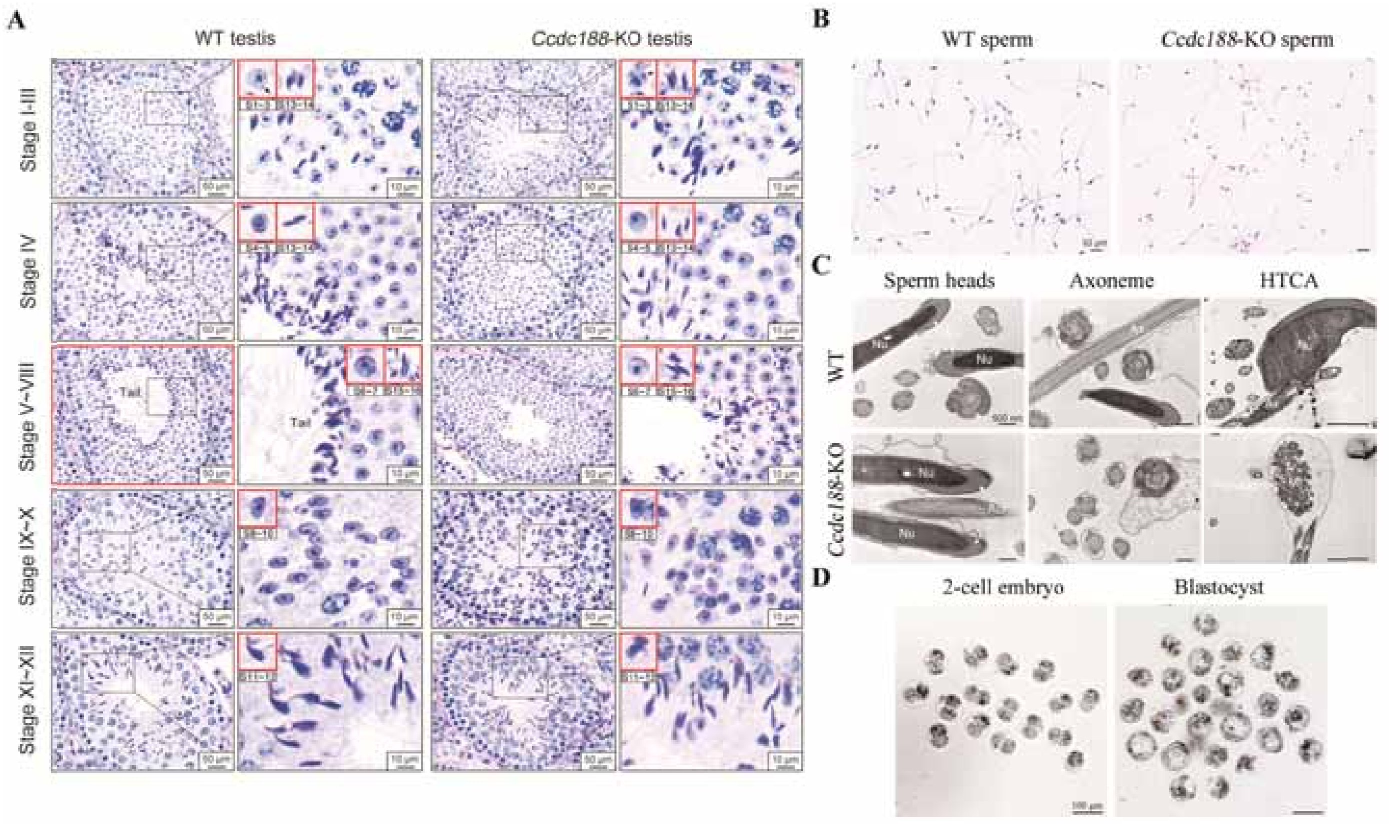
A
WT testis
Ccdc188-KO testis
Stage I-III
Stage IV
Stage V~VIII
Stage IX~X
Stage XI~XII
Tail
B
WT sperm
Ccdc188-KO sperm
C
Sperm heads
Axoneme
HTCA
WT
Ccdc188-KO
Nu
D
2-cell embryo
Blastocyst

Figure S3.

Supplementary Table Legends

Table S1. Proband's information and his semen characteristics.

Table S2. Candidate pathogenic variations identified in the proband by WES and analysis.

Table S3. CCDC188-interacting proteins in mouse testis protein lysates identified by IP-MS.

Table S4. Primers used for the verification of the c.937C>T variant affecting the human *CCDC188* gene.

Table S5. The sgRNA sequence to generate *Ccdc188*-KO mouse strain.

Table S6. Primers for plasmid construction.

Table S7. All antibodies used in this study.

Video S1. The motility and morphology of sperm from WT mice.

Video S2. The motility and morphology of sperm from *Ccdc188*-KO mice.

Table S1. Proband's information and his semen characteristics.

Subject	IV-1	
Age	30 years	
Physical examination	normal	
Karyotype	46, XY	
Y Chr micro-deletion	no	
	Semen Parameters	Reference limits
Volume (ml)	2.7	>1.5
Concentration (10^6/ml)	2.77	>15
Motility (%)	24.4	>40
Progressive motility (%)	14.64	>32
Morphological normality (%)	0	>4
Acephalic sperm (%)	100	

Lower reference limits for semen analysis are provided according to the World Health Organization (WHO) standards. At least 200 spermatozoa were observed for morphology analysis.

Table S2. Candidate pathogenic variations identified in the proband by WES and analysis.

Gene	Type	Variation	ExAC_ALL	ExAC_EAS	SIFT	Polyphen2	MutationTaster	CADD	GERP++	phyloP100way	Inherited pattern
ANKRD2	nonsynonymous SNV	ANKRD2:NM_001291218 c.C17G:p.P6R	.	.	.	.	.	.	.	.	homozygous
CCDC188	**stopgain**	**CCDC188:NM_001365892.2 c.C937T:p.R313***	**.**	**.**	**.**	**.**	**.**	**40**	**3.79**	**0.907**	**homozygous**
CCND2	nonsynonymous SNV	CCND2:NM_001759 c.C64G:p.R22G	2.58E-05	0.0004	T	B	N	14.83	3.09	-0.191	homozygous
FAT4	nonsynonymous SNV	FAT4:NM_001291285 c.A3067G:p.K1023E	4.14E-05	0.0006	D	B	D	13.9	5	2.145	homozygous
FOXD4L4	nonsynonymous SNV	FOXD4L4:NM_199244 c.G839C:p.G280A	.	.	T	.	N	2.608	2.18	-0.517	homozygous
GGT2	nonsynonymous SNV	GGT2:NM_001351304 c.C1664T:p.T555M	.	.	T	.	N	12.67	1.68	2.398	homozygous
GOLGA6L9	nonsynonymous SNV	GOLGA6L9:NM_198181 c.C451G:p.Q151E	.	.	T	.	.	5.077	0.046	0.047	homozygous
ITPRIPL2	nonsynonymous SNV	ITPRIPL2:NM_001034841 c.G1462C:p.A488P	.	.	T	B	N	12.53	5.22	0.01	homozygous
KIAA1024	nonsynonymous SNV	KIAA1024:NM_015206 c.C862T:p.P288S	8.24E-06	0	D	.	D	1.481	5.29	0.763	homozygous
LOC100129307	nonsynonymous SNV	LOC100129307:NM_001310140 c.C446T:p.T149M	.	.	.	.	.	.	.	.	homozygous
MAN2C1	nonsynonymous SNV	MAN2C1:NM_001256496 c.A1040G:p.N347S	1.93E-05	0.0003	T	D	D	23.7	5.54	7.434	homozygous

N4BP2	nonsynonymous SNV	N4BP2:NM_018177 c.T2438C:p.V813A	.	.	T	P	N	11.36	5.64	0.306	homozygous
NR2E3	nonsynonymous SNV	NR2E3:NM_014249 c.T809C:p.L270P	.	.	D	P	.	28.3	5.36	7.911	homozygous
RLBP1	nonsynonymous SNV	RLBP1:NM_000326 c.C107T:p.P36L	.	.	T	B	N	0.417	5.95	-0.305	homozygous
DPP7	nonsynonymous SNV	DPP7:NM_013379 c.G1177A:p.D393N	0.0003	0	T	B	N	14.14	4.18	0.289	compound heterozygous
DPP7	nonsynonymous SNV	DPP7:NM_013379 c.G658A:p.V220M	8.29E-06	0.0001	D	D	D	24.7	5.11	5.931	compound heterozygous
SPATA31C1	frameshift insertion	SPATA31C1:NM_001145124 c.224_225insGC:p.C76Rfs*13	7.57E-05	0.001	.	.	.	.	.	.	compound heterozygous
SPATA31C1	stopgain	SPATA31C1:NM_001145124 c.C1381T:p.Q461*	.	.	.	.	.	.	.	.	compound heterozygous
GPR50	nonsynonymous SNV	GPR50:NM_004224:exon2:c.C928T:p.H310Y	1.15E-05	0.0002	D	P	N	20.6	4.63	1.308	hemizygous

Table S3. CCDC188-interacting proteins in mouse testis protein lysates identified by IP-MS.

Protein Name	Peptide	Abundance CCDC188-IP(1)	Peptide	Abundance IgG-IP(1)	Peptide	Abundance CCDC188-IP(2)	Peptide	Abundance IgG-IP(2)
ABCF1	4	1388714.447	3	95212.32778	2	4242901.428	1	0
AGO2	2	152655.7797	1	0	2	1154051.555	1	0
AP2S1	2	3614877.672	1	0	1	503894.9781	1	0
BCLAF1	5	4523194.505	1	0	3	1293743.994	1	0
BUD31	2	564821.0144	1	0	3	1425254.251	1	0
CAPZA1	3	1642419.848	1	0	2	194774.4048	2	0
CASC3	2	992918.7054	1	49575.63559	2	58623343.77	1	80216.45187
CATSPER1	2	1011079.361	1	0	2	849897.0004	1	0
CCAR1	8	4031249.405	2	0	7	660382.3945	1	0
**CCDC188**	**2**	**1311869.365**	**1**	**0**	**5**	**970548.8768**	**1**	**0**
CDC5l	11	13104827.84	1	0	9	5675866.507	1	544681.9764
CHTOP	6	2088517.395	1	0	6	1981336.724	1	0
CNTNAPP3	1	7504209.564	1	0	1	16817706.89	1	12532122.54
COIL	25	74678611.97	1	0	18	64396007.26	1	0
CPEB4	1	179245.8426	1	0	1	124840.073	1	0
CPSF4	1	304545.9061	1	0	2	299900.1592	1	0
CSNK2A1	7	7487280.357	1	0	7	1188198.685	1	0
CTC1	1	7126.675361	1	0	1	119080.5303	1	0
DBT	7	23089144.84	1	0	4	5967474.351	1	0
DDX23	4	7428883.373	1	0	2	4665188.072	1	0
DECR1	1	698217.1721	1	0	2	1007580.592	1	0
DENN2B	1	946053.046	1	0	1	2070328.942	1	0

DHX36	6	2821613.272	1	0	4	673117.3347	1	0
DKC1	10	49468626.68	1	0	8	7330207.531	1	0
DLAT	37	133926680.2	22	13291925.2	35	83634984.63	12	5588679.666
DNAJB11	1	313881.6014	1	0	1	673732.2995	1	0
DPYSL3	5	1415346.783	1	0	4	1500579.44	1	0
DTYMK	1	649279.5851	1	0	1	2340462.089	1	0
EIF3K	7	4499377.994	3	0	6	7280284.257	1	231859.0056
ELAVL2	5	3199344.953	1	0	7	2712844.591	1	0
FAM98A	10	20932272.25	10	771418.8971	10	15769718.88	11	6561275.143
FBL	15	19560429.96	1	0	12	12974756.64	1	0
FHL4	4	1466455.608	1	0	1	34210.48065	4	0
FKBP6	4	3313521.278	1	0	3	207024.0283	3	0
FYTTD1	7	108492461	2	487195.2845	11	147115892.1	2	582177.0824
GAPDH	23	73160299.54	1	0	20	27468271.89	1	0
GAR1	2	2461746.249	1	0	3	3653853.045	1	0
GEMIN2	1	912739.3267	1	0	3	2551417.666	1	0
GLYR1	3	728294.8015	1	0	3	2065617.606	1	149396.1657
GM1141	25	32951978.33	1	234780.2727	22	22641669.26	1	0
GSTO2	2	1090700.783	1	0	1	989636.9187	2	0
GTPBP1	3	8753471.45	1	0	1	347202.2042	1	0
H2BC1	8	10613130.87	1	0	2	513512.2374	1	0
HILS1	6	7043675.222	1	0	4	5300687.173	1	0
HMGCLL1	1	1199491.484	1	0	1	1470839.176	1	0
HNRNPH3	19	100031969.2	1	0	22	53859620.15	1	0
HNRNPK	50	267225348.2	1	0	45	262606491.6	1	0
HNRNPR	14	12008038.34	1	0	10	6668056.089	1	0

HPX	1	29631.93889	1	0	4	180530.1379	1	0
HRG	3	623449.5713	1	62296.35924	2	476764.3523	1	0
HTRA2	4	43935450.43	1	0	1	424820.9758	1	0
IFT122	7	2875746.477	1	0	10	6387300.101	1	0
IFT140	7	913972.529	1	0	8	4255185.626	1	0
IGF2BP3	4	1304967.848	1	0	2	481196.6281	1	0
IQCG	2	35125311.15	2	1538288.956	1	486148.4956	1	0
IQCN	1	3001302.774	1	0	1	436814.0653	1	0
JCHAIN	6	9494430.946	1	132742.6217	6	9493600.46	1	0
KIF27	2	3466099.229	1	0	1	505793.0349	1	0
KLHL36	1	15777.63314	1	0	3	555947.6181	1	0
LACTB	18	14893899.07	3	583953.9275	13	25521156.75	3	233032.2075
LARP4	1	264877.9544	1	0	2	3004954.942	1	0
LASP1	2	626777.5108	1	0	2	537715.17	1	0
LIG3	8	3532905.85	1	0	5	224741.4296	1	0
LSM14B	4	449602.1851	1	0	5	470322.3384	3	0
MAGOHB	7	9245064.217	1	0	4	7363417.545	2	190003.6757
MEIOC	5	397919.7256	1	0	2	1597310.738	1	0
MFAP1B	5	37961019.02	3	449290.234	2	6363759.947	2	98546.5408
MLF1	1	450345.848	1	0	1	822575.4188	1	0
MTDH	2	712844.2105	1	0	1	414068.8639	1	0
MUG1	3	2539508.791	1	0	4	505482.0366	1	0
MVP	18	9884304.164	1	0	14	11250573.34	14	2824185.32
NCBP2	3	3308632.088	1	0	4	5788926.661	1	0
NCL	17	24813675.37	1	0	13	15361437.67	1	0
NCOA5	31	138627281.9	9	3520745.787	22	234800770.2	8	2400827.061

NOP56	21	42735640.67	1	0	11	31085690.62	1	0
NUDT16L1	5	1776107.18	1	0	6	2455243.722	1	0
NUFIP2	5	9830345.793	1	0	2	1169484.921	1	0
PAFAH1B1	1	20081060.92	1	0	1	462486.0123	1	0
PDHA1	1	8085309.043	1	0	2	9281603.441	1	0
PDHX	12	13212527.5	4	1265162.762	8	6877111.228	2	561136.9601
PDZD8	6	391487.3298	1	0	8	3940606.762	1	0
PGAM5	8	11300069.41	2	218149.5239	8	19177670.66	6	784162.1291
PGM5	1	356234.9107	1	0	1	917687.3339	1	0
**PMFBP1**	**11**	**4609621.473**	**1**	**0**	**15**	**5147362.322**	**1**	**0**
PNN	5	1066786.492	1	0	7	1461687.601	1	0
PPIL1	1	3005002.991	1	0	1	3213788.856	1	0
PPP1CC	1	339867.8999	3	0	1	226285.0025	1	0
PPP2R2A	2	6776403.406	1	0	2	3416480.747	1	0
PRM2	1	947267.2122	1	0	1	563387.8363	1	0
PRPF3	1	148635.2907	1	0	1	404123.2916	1	0
PRPF40A	4	17708661.24	1	60893.14595	1	142862.0523	1	0
PRPS1L1	1	112551.7808	1	0	1	321106.2213	1	0
PSIP1	4	10629084.9	1	0	2	426408.1445	1	0
PSMA6	1	68843.09642	1	0	3	611094.3895	1	0
PSMG1	1	145836.0981	1	0	1	688543.5786	1	68366.39034
PUM1	1	198427.8182	1	0	2	796560.564	1	0
PWWP3A	6	863395.8481	1	0	2	43362711.76	1	309944.7683
PZP	5	14163551.23	1	0	6	10883084.66	1	0
QSOX2	1	2438732.843	1	0	1	1333546.842	1	0
RAB1A	3	5287063.235	1	0	2	2438782.327	1	0

RALY	5	1490250.323	1	0	7	2494974.706	1	0
RBM46	17	29198159.46	5	987857.2484	13	13278172.51	4	324515.995
RBMXL1	8	7869096.992	1	0	7	4973063.485	1	0
RBX1	2	1312561.511	1	0	1	400921.1939	1	0
RDH11	3	1800705.155	1	121969.9877	4	34152717.55	4	740828.9022
RNF151	1	297670.777	1	0	1	384138.6274	1	36055.14448
RPL12	6	17086516.54	1	0	10	27267234.37	1	0
RPL13A	9	1709396134	1	0	9	928160939.1	1	0
RPL18	5	18327699.07	1	0	5	158696671.4	1	0
RPL18A	4	2395504.919	3	235627.5863	5	6244483.778	3	269079.9889
RPL21	2	3936175.5	1	0	5	7863615.289	1	0
RPL23A	2	1779485.162	1	0	3	8254981.314	1	0
RPL27	4	3020409.469	1	0	4	9443284.84	1	0
RPL28	1	359613.6991	1	0	2	1629887.649	1	0
RPL32	1	433734.7537	1	0	1	322588.5188	1	0
RPL34	3	3109504.403	1	0	2	2507421.252	1	0
RPL37A	1	11750249.53	1	0	1	7712229.507	1	0
RPL6	10	16094560.98	1	0	8	7124427.354	1	0
RPL7A	16	20085037.26	4	684312.9651	11	24206876.79	4	1827427.537
RPL8	7	43839028.01	1	0	6	63474106.51	3	4228464.973
RPL9-PS6	13	15349486.24	1	0	12	5457869.069	4	43725.33374
RPLP1	1	434131.8748	1	0	1	701211.9641	1	0
RPLP2	5	1455154.89	1	0	4	4326705.821	1	0
RPS13	5	6220625.358	3	520356.1044	8	11952028.44	2	361744.4508
RPS15A	7	12594951.82	4	502262.8264	10	15482089.4	5	1244454.801
RPS23	1	2152244.538	1	51042.73157	3	3607101.796	1	159216.9142

RPS26	1	1001845.477	1	0	2	3183766.785	1	0
RPS4X	5	4987788.016	1	174202.9759	5	4461532.609	1	362507.8743
RPS6	7	8830206.678	3	720345.5958	10	13207012.48	1	0
RPS8	8	17963135.67	1	0	8	18867533.84	1	0
SAMD4B	1	485912.5797	1	0	1	208032.9873	1	0
SERPING1	3	3183204.49	1	0	5	2507668.953	1	0
SLTM	4	2045135.06	1	0	2	795795.2605	1	0
SNRPA	2	1173479.517	1	0	2	1326032.904	1	0
SNRPA1	8	5972935.128	1	0	7	6738725.906	1	0
SNRPD2	9	8216900.039	1	0	6	8787656.622	1	0
SNRPD3	3	2576075.644	1	152678.062	3	5173862.61	1	205003.6296
SON	3	3513074.907	1	0	1	489896.8398	1	0
SPAG4	5	3896083.305	1	0	3	517147.6851	1	0
SPATS2	6	12724164.34	2	121736.5245	6	1365592.534	1	0
SRSF5	6	3809745.394	1	0	5	3660262.477	1	0
SRSF6	3	3227869.177	1	0	4	7892027.259	1	0
SRSF9	5	1250157.582	1	47814.80489	3	1144369.911	1	0
SSX2IP	1	279384.7636	1	0	2	254050.5293	1	0
STAU1	4	5891621.212	1	0	4	2279474.875	1	115330.1248
STK39	1	398296.7047	1	0	1	212692.9631	1	0
STRBP	6	1500736.938	1	0	5	2479776.339	1	0
SUGP2	36	20342484.2	11	1095000.184	36	21366997.03	3	845301.3373
**SUN5**	**3**	**6093713.452**	**1**	**0**	**4**	**24981983.37**	**1**	**0**
TARBP2	7	9782645.082	1	0	6	3663424.378	1	0
TDRD3	4	3875700.652	1	0	3	1589801.325	1	0
TEKT1	5	1290149.515	1	0	3	417467.9546	2	0

TEX13D	1	6834964.343	1	0	2	8536619.257	1	0
TEX30	1	972808.1865	1	0	1	1366147.708	1	0
TEX35	1	471337.2235	1	0	2	1049916.599	1	0
THOC2	3	283097.2048	1	0	2	238613.4259	1	0
THOC6	1	373626.9266	1	0	1	1177110.808	1	0
TMCO5	2	2172693.364	1	0	3	2471815.266	1	0
TMEM191C	4	9591313.932	1	0	6	9698863.584	1	0
TNRC6A	1	221779.5289	1	0	1	2278614.358	1	0
TOP2A	4	1194894.309	1	0	1	430349.9613	1	0
TOP3B	6	13211713.25	1	0	5	11632956.29	1	0
TRA2B	6	25190015.05	3	741911.0002	7	18178421.99	3	1660932.467
TRIM14	1	152146.2417	1	0	1	128102.3724	1	0
UPF2	3	494992.961	1	0	4	994106.6528	1	0
USP10	2	182243.6571	1	0	4	1184864.954	1	19536.17847
USP40	2	263460.1962	1	0	2	1013373.381	1	0
VTN	3	2272054.928	1	0	2	780043.4855	1	0
WDR33	10	107956646.5	4	2856365.058	6	50851086.26	1	0
WDR5	3	33570774.81	1	0	1	25061300.67	1	0
WRAP53	4	374687.0033	1	0	3	1979958.234	1	0
ZFP748	1	3563383.615	1	0	1	3278320.299	1	0
ZNF326	30	25456624.14	1	0	19	30212400.01	1	0
ZNFX1	5	1558483.315	1	0	7	2371029.779	1	0

Table S4. Primers used for the verification of the c.937C>T variant affecting the human *CCDC188* gene.

Variant	Sequence (5'→3')	Size
c.937C>T/p.R313*	CTGCTGTGCTTACCTCCTCTGG<br>CAGGAAGCCGTCCACTTTGAG	883 bp

Table S5. The sgRNA sequence to generate *Ccdc188*-KO mouse strain.

Variant	Sequence (5'→3')	PAM
gRNA-1<br>gRNA-2	ATCCCTGACAGGGCTGATCC<br>GTCCACTTCGAGGCGCAAGA	AGG

Table S6. Primers for plasmid construction.

Name	Sequence (5'→3')	Size
SUN5	(KpnI) CGGGGTACCCCGATGCCCCGGACGAGGAAC<br>(XbaI) TGCTCTAGAGCAGGATAGGGGCTCTAGGTGTGA	1047 bp
PMFBP1	(KpnI) CGGGGTACCCCGATGCTAAAGCTTAAAGGAGAATT<br>(XbaI) TGCTCTAGAGCAGCAGTAGGAGGAGCCTGAG	3069 bp
CCDC188	(HindIII) CCCAAGCTTGGGATGGAGGGGCCAAAACCC<br>(EcoRI) CCGGAATTCCGGGAAAGGCAGGAAGTCGTCCACT	1308 bp

Table S7. All antibodies used in this study.

Name	Factory	Catalog	Application
CCDC188 polyclonal antibody	Home-made		WB (1:1000), IF (1:200)
SUN5 polyclonal antibody	Proteintech	17495-1-AP	WB (1:1000), IF (1:200)
PMFBP1 polyclonal antibody	Proteintech	17061-1-AP	WB (1:1000), IF (1:200)
β-actin monoclonal antibody	Abcam	ab8226	WB (1:1000)
ac-TUB monoclonal antibody	Proteintech	66200-1-Ig	IF (1:200)
ac-TUB monoclonal antibody	CST	#5335	IF (1:200)
Flag-tag monoclonal antibody	Abmart	M20008	IP (1:100), WB (1:2000)
Myc-tag monoclonal antibody	Abmart	M20002	IP (1:100), WB (1:2000)
Goat Anti-Rabbit IgG-HRP	Abmart	M21002	WB (1:5000)
Goat Anti-Mouse IgG-HRP	Abmart	M21001	WB (1:5000)
Alexa Fluor 488-labeled Goat Anti-Rabbit IgG(H+L)	Beyotime	A0423	IF (1:200)
Alexa Fluor 555-labeled Donkey Anti-Mouse IgG(H+L)	Beyotime	A0460	IF (1:200)
Alexa Fluor 488-labeled Goat Anti-Mouse IgG(H+L)	Beyotime	A0428	IF (1:200)
Alexa Fluor 555-labeled Donkey Anti-Rabbit IgG(H+L)	Beyotime	A0453	IF (1:200)
